# Effects of the number of removed lymph nodes on survival outcome in patients with sentinel node-negative breast cancer

**DOI:** 10.1186/s12957-021-02418-9

**Published:** 2021-10-19

**Authors:** Calogero Cipolla, Antonio Galvano, Salvatore Vieni, Federica Saputo, Simona Lupo, Mario Latteri, Giuseppa Graceffa, Maria Rosaria Valerio

**Affiliations:** Department of Surgical, Oncological and Oral Sciences, University of Palermo (Italy), A.O.U.P. “P. Giaccone” University Hospital, Via del Vespro 129, 90127 Palermo, Italy

**Keywords:** Breast cancer, Axillary staging, Sentinel lymph node biopsy, Optimal number, False-negative rate, Survival, Prognosis

## Abstract

**Background:**

Sentinel lymph node biopsy is the gold standard surgical technique for axillary staging in patients with clinically node-negative. However, it is still uncertain what is the optimal number of sentinel lymph nodes (SLNs) to be removed to reduce the false-negative rate. The aim of this study was to investigate whether patients with a single negative SLN have a worse prognosis than those with two or more negative SLNs.

**Methods:**

A retrospective review was conducted on a large series of SLN-negative breast cancer patients. Survival outcomes and regional recurrence rate were evaluated according to the number of removed SLNs. Secondly, the contribution of different adjuvant therapies on disease-free survival was explored. Statistical analysis included the chi-square, Wilcoxon–Mann–Whitney test, and Kaplan–Meier survival analysis.

**Results:**

A total of 1080 patients were included in the study. A first group consisted of 328 patients in whom a single SLN was retrieved, and a second group consisted of 752 patients in whom two or more SLNs were retrieved. There was no relevant difference in median DFS (64.9 vs 41.4) for SLN = 1 vs SLN > 1 groups (HR 0.76, *CI 95%* 0.39–1.46; *p* = 0.38). A statistically significant difference in mDFS was showed only for HT-treated patients who were SLN = 1 if compared to SLN > 1 (100.6 months versus 35.3 months).

**Conclusions:**

There is likely a relationship between the number of resected SNL and mDFS. Our results, however, showed no relevant difference in median DFS for SLN = 1 vs SLN > 1 group, except for a subset of the patients treated with hormone therapy.

## Background

For two decades now, sentinel lymph node biopsy (SLNB) has replaced axillary lymph node dissection (ALND) as the standard minimally invasive procedure for axillary staging in clinically node-negative (cN0) breast cancer. Some important clinical trials demonstrated that SLNB alone is equivalent to ALND in axillary staging, with fewer associate postoperative morbidity outcomes. Moreover, SLNB alone found equivalent efficacy to upfront ALND in disease-free survival (DFS), overall survival (OS), and regional recurrences (RR) [1, 2].

However, despite many studies subsequently confirmed these findings [3], some concerns remain regarding the false-negative rate (FNR) associated with the use of SLNB alone. Weaver et al. [4] reported a 15.9% incidence of occult metastases after further examination of initially pathologically negative sentinel lymph nodes (SLNs). In this study, patients with occult metastasis had worse OS, DFS, and distant DFS than those without occult nodal metastasis. Moreover, some other authors confirmed an increase of false-negative rates if a single SLN has been removed [5, 6]. Ban et al. [7] reported a FNR of 26.6% when a single SLN was retrieved, compared to 0% for four or more nodes, suggesting that four SLNs may represent an optimal threshold. The count did not include the parasentinel lymph nodes. Other authors conclude that at least two SLNs may be removed to reduce the FNR and to improve the outcomes in terms of OS, DFS, and RR [8, 9].

Based on the aforementioned studies, it could be concluded that missed positive nodes may lead to an understaging and thus to undertreatment of those SLN patients with occult metastasis within the SLNB. It also indicates a decreased negative predictive value for SLNB in settings where only a single SLN is detected. However, none of these studies investigated the outcomes in terms of OS, DFS, and local and distant recurrences for patients with a single SLN comparing them with those of patients with two or more SLNs assessed.

Based on these assumptions, the main purpose of our study was to investigate whether patients in whom only one lymph node was found during SLNB have a worse prognosis than patients in whom two or more sentinel lymph nodes were found and examined.

## Materials and methods

After approval by the Institutional review board at University Hospital “AOUP Paolo Giaccone” of Palermo, we collected and retrospectively analyzed the medical records of a large series of patients with primary cN0 invasive breast cancer observed at our Institution from January 2013 to September 2019. Each patient underwent synchronous excision of the breast cancer either by conservative surgery or by total mastectomy and SLNB. Whole breast radiation therapy and systemic adjuvant therapies were administered according to the international guidelines.

Eligibility criteria were a preoperative diagnosis of primary invasive breast cancer defined through fine needle aspiration cytology and/or by percutaneous needle core biopsy, clinically negative ipsilateral axillary lymph nodes (cN0), and pathologic negative axillary lymph nodes (pN0) at SLNB. Exclusion criteria from the study were clinically positive ipsilateral axillary lymph nodes, a previous neoadjuvant therapy, pathologic positive axillary lymph nodes at SLNB, inflammatory breast cancer, locally recurrent breast cancer, metastatic disease at the diagnosis, and lack of follow-up data.

SLN was detected using the identification technique with radiotracer and, if necessary, the use of the vital dye, as described in our previous studies [10–13]. Briefly, all patients underwent a preoperative lymphoscintigraphy employing a subdermal periareolar injection of 99Tc-labeled human albumin colloid (10–12 MBq di Tc-99m in 0.2 ml of albumin colloid), 18–24 h before surgery. For intraoperative identification of the SLN, a radio-guided surgical probe was used to identify the area with the greatest radioactive intensity. Limited to the cases were the radio-guided surgical probe detected a weak radiotracer signal, a subareolar injection of 0.5–0.8 ml of vital stain about 10–15 min before surgery was performed.

All hot and/or blue lymph nodes were removed and submitted immediately for intraoperative assessment by frozen section (FS). Intraoperative examination was carried on two 4-μm frozen sections stained with hematoxylin and eosin (H&E). All the remaining tissue was formalin-fixed, paraffin-embedded, and entirely sectioned at 100-micron intervals for the definitive histopathological examination. Finally, any enlarged palpable or suspect lymph nodes were removed and sent for definitive histopathological examination to reduce the risk of a false negative at SLNB due to abnormal lymphatic drainage.

All patients with SLN macro-metastasis at the frozen section underwent immediate completion ALND. In the cases of SLN negative at frozen section but positive for macrometastases at definitive histopathological examination, patients underwent a delayed completion ALND. No completion ALND was performed in women with metastasis-free SLN, ITC, and patients with SLN micrometastases, either at intraoperative or at final histopathological examination. Several studies have actually shown that in patients where SLN is positive for micrometastasis, ALND can be safely omitted because it does not improve survival [14–17].

The primary endpoint was to evaluate the median disease-free survival (mDFS) according to the number of nodes collected after SLNB (1 or more than 1). Secondly, we would explore the contribution of different adjuvant therapies: hormone therapy (HT), hormone therapy plus chemotherapy (HT+CT), or chemotherapy (CT) on mDFS. Steroid or non-steroidal aromatase inhibitors have been used in postmenopausal patients, while tamoxifen plus LH-RH analogs in premenopausal patients. Furthermore, we would evaluate the regional recurrences (RRs) and the distant metastases (DM) rates according to SNLB (1 or more than 1). RRs include both local intramammary and axillary recurrences.

We analyzed all patients according to the age at the diagnosis, type of surgery, SLN status, tumor size, histological type, estrogens (ER) and progesterone (PR) receptor status, and human epidermal growth factor receptors 2 (HER2) status. Tumors with ≥1% positive nuclear-stained cells were considered positive for ER and PR expression, and those with an immunohistochemical score +3 according to the ASCO/CAP 2018 criteria [18] were considered positive for HER2. Moreover, radiation therapy and systemic adjuvant therapy administration were evaluated as co-variables to determine whether in each of these patients the tumor or associated treatment was predictive factors for recurrence and/or death.

The patients included in the study based on the above-mentioned criteria were divided into two groups according to the number of negative SLNs found intraoperatively. The first group included patients in whom SLNB has found a single negative SLN, while the second group included all patients with two or more negative SLNs. The distributions of patients in groups with respect to baseline demographic and clinical characteristics will be compared through the chi-square test for heterogeneity and the Wilcoxon–Mann–Whitney test for categorical and continuous variables, respectively. Survival (DFS and OS) analysis will be performed using the Kaplan–Meier method, providing median and *p* values, with the use of the log-rank test for comparisons. Outcomes with a *p* value < 0.05 will use as a threshold for statistical significance. All the statistical analyses will be performed using SPSS statistics software, version 20 (IBM, Armonk, NY, USA) [19].

## Results

In the period between January 2013 and September 2019, a total of 1901 women with primary cN0 invasive breast cancer underwent a sentinel lymph node biopsy at our Surgical Oncology Unit, according to the aforementioned inclusion criteria.

At intraoperative FS examination of the SLNs, macrometastases were found in 281 patients (14.8%) and micrometastases in 18 patients (0.9%), whereas 1602 patients (84.3%) had negative SLN. However, SLN metastases were found in 181 cases (9.5%) at the definitive histopathological examination, increasing the number of patients with positive SLNs to a total of 480 (25.2%). These patients were excluded from the study. Therefore, 1422 patients with a confirmed diagnosis of negative SLNs were considered eligible for the study. However, a further 342 of these were excluded from the study due to missing data.

**Fig. 1:**
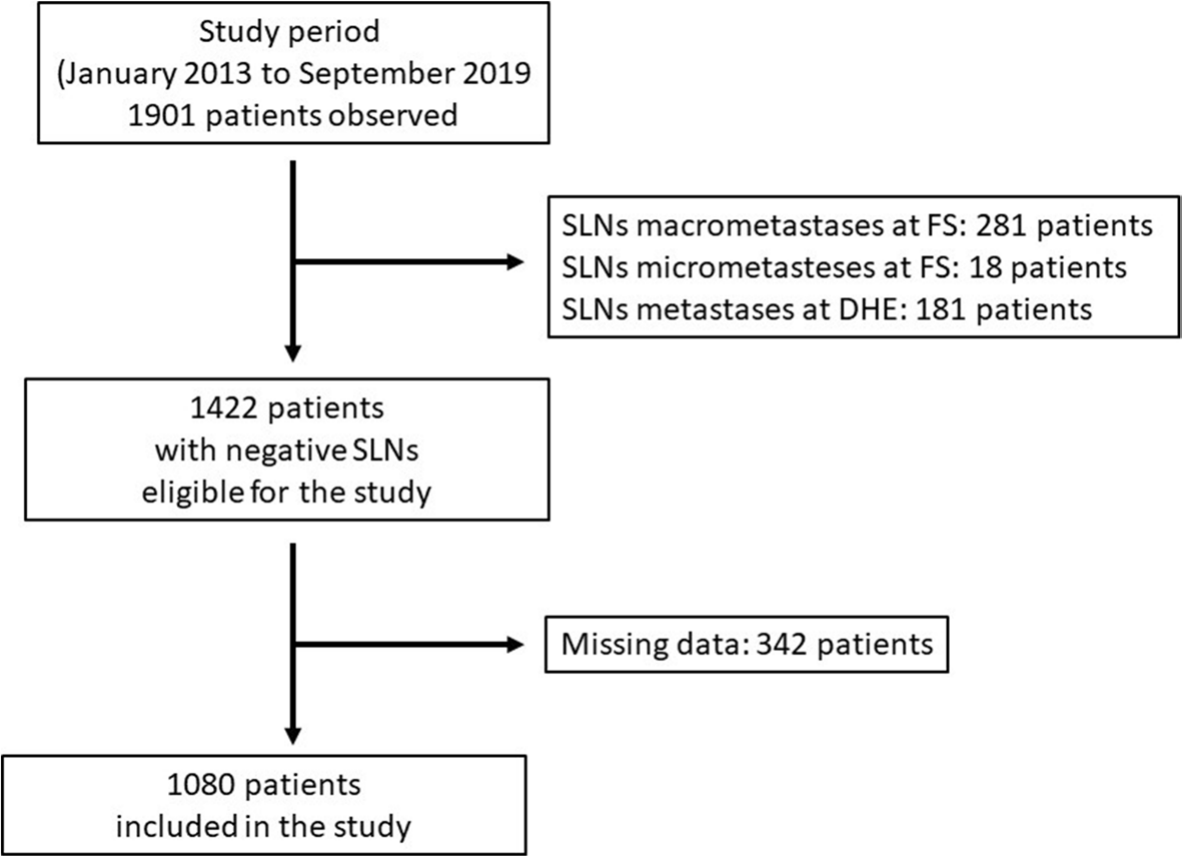
Flow chart of patients with negative SLNs. SLNs sentinel lymph nodes, FS frozen section, DHE definitive histopathological examination

Finally, a total of 1080 patients were included in the study (Fig. 1). The first group consisted of 328 patients in whom a single SLN was retrieved, and the second group consisted of 752 patients in whom two or more SLNs were retrieved.

The average number of negative SLN removed in this group was 2.1 (range 1–10 lymph nodes). The median follow-up ranged from 6 to 72 months with a median of 44.5 months.

The demographic and clinical-pathological characteristics of the tumor are summarized in Table 1.

**Table 1:** Demographic and clinical-pathological characteristics of the patients

	SLN = 1 *n*. 328		SLN >1 *n*. 752		*P*-value
	*n.*	%	*n.*	%	
**Age** (years)					
<40	12	3.6%	20	2.7%	0.91
40–49	56	17%	112	15%	
50–69	216	66%	524	69.6%	
>70	44	13.4%	96	12.7%	
**Tumor size**					
T1(<20mm)	216	65.8%	536	71%	0.45
T2 or greater	112	32.9%	216	29%	
**Grading**					
I	96	29.3%	196	26%	0.95
II	156	47.6%	384	51%	
III	52	15.8%	116	15.5%	
Indeterminate	24	7.3%	56	7.5%	
**Histology**					
Invasive ductal	240	73.2%	568	75.5%	0.91
Invasive lobular	36	10.9%	72	9.6%	
Others	52	15.9%	112	14.9%	
**Receptor status**					
ER+	294	89.6%	660	87.7%	0.79
PR+	270	82.2%	605	80.5%	
HER2/neu+	107	32.7%	240	31.9%	
ER+, PR+, HER2+	75	22.8%	168	22.4%	
Triple negative	13	4.1%	49	6.5%	

The results of our study first confirm the prognostic role of T stage on disease recurrence/relapse (T1 = 1.8%; T2 2.4%, T3 = 10.5% - chi-square 12.9; *p* = 0.002) regardless of the number of removed SLNs. No statistically significant differences were provided in SNL = 1 and SNL > 1 cohort according to different relevant parameters (age, tumor size, grading, histology, receptor status). Furthermore, no difference was also underlined in cancer treatments and in particular, surgery (total mastectomy or quadrantectomy), radiant therapy, and systemic therapy (HT, HT+CT, or CT). Of note, the large majority of our population received conservatory strategy except for rare cases associated with very large, multicenter, or aggressive tumor features (Table 2). In all patients treated with conservative surgery, local radicality was always confirmed by “no ink on tumor” resection margins.

**Table 2:** Cancer treatment

	SLN = 1 *n*. 328		SLN > 1 *n*. 752		*P* value
	*n*.	%	*n*.	%	
Surgery					0.06
Quadrantectomy	328	100%	736	7.9%	
Total mastectomy	___		16	2.1%	
Radiant therapy	328	100%	736	97.9%	0.61
Systemic therapy					0.99
Chemotherapy	20	6%	71	9.5%	
Hormone therapy	244	74.3%	551	73.3%	
Combination therapy	64	19.5%	130	17.2%	

In our enrolled population, a total of 17 RRs were registered during the follow-up period. In particular, 6 RRs were in the SLN = 1 cohort and 11 RRs in SLN > 1 cohort respectively. Our series reported a lower percentage of distant relapses around 0.8% (0.6% visceral; 0.2% bone). Four DM were in the SLN = 1 group while 7 DM were in the SLN >1 group. Table 3 underlines tumor recurrences, local and distant, in both patient groups. Through a detailed comparison between the local and remote recurrence rates, we notice a greater number of local recurrences in the group with two or more negative sentinel lymph nodes intraoperatively, and, conversely, more remote recurrences in the group with a single negative sentinel lymph node. It is not, however, a statistically significant association.

**Table 3:** Regional and distant tumor recurrences

Recurrences	SLN = 1 *n*. 328		SLN > 1 *n*. 752		*P* value
	*n*.	%	*n*.	%	
Total	10	3.0%	18	2.4%	0.98
Free-disease patients	316	97.0%	724	97.6%	
Recurrence sites					
Local	6	1.8%	11	1.5%	0.35
Distant	4	1.2%	7	0.9%	0.17

**Fig. 2:**
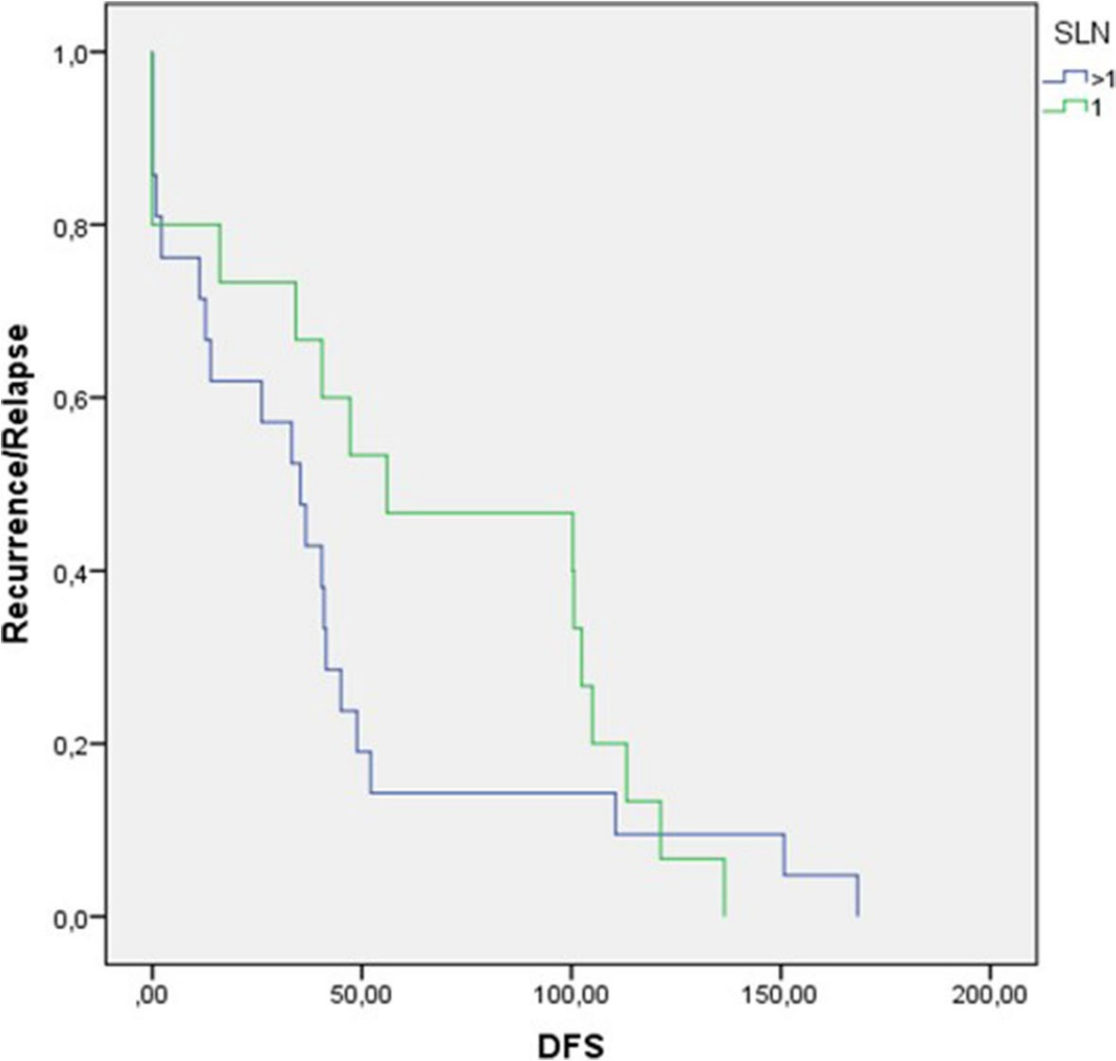
Kaplan–Meier showing median disease-free survival results according to sentinel lymph nodes

Besides, overall population mDFS was 40.4 months. Our results showed no relevant difference in median DFS (64.9 vs 41.4) for SLN = 1 vs SLN > 1 group (*HR* 0.76, *CI 95%* 0.39–1.46; *p* = 0.38) (Fig. 2).

**Fig. 3:**
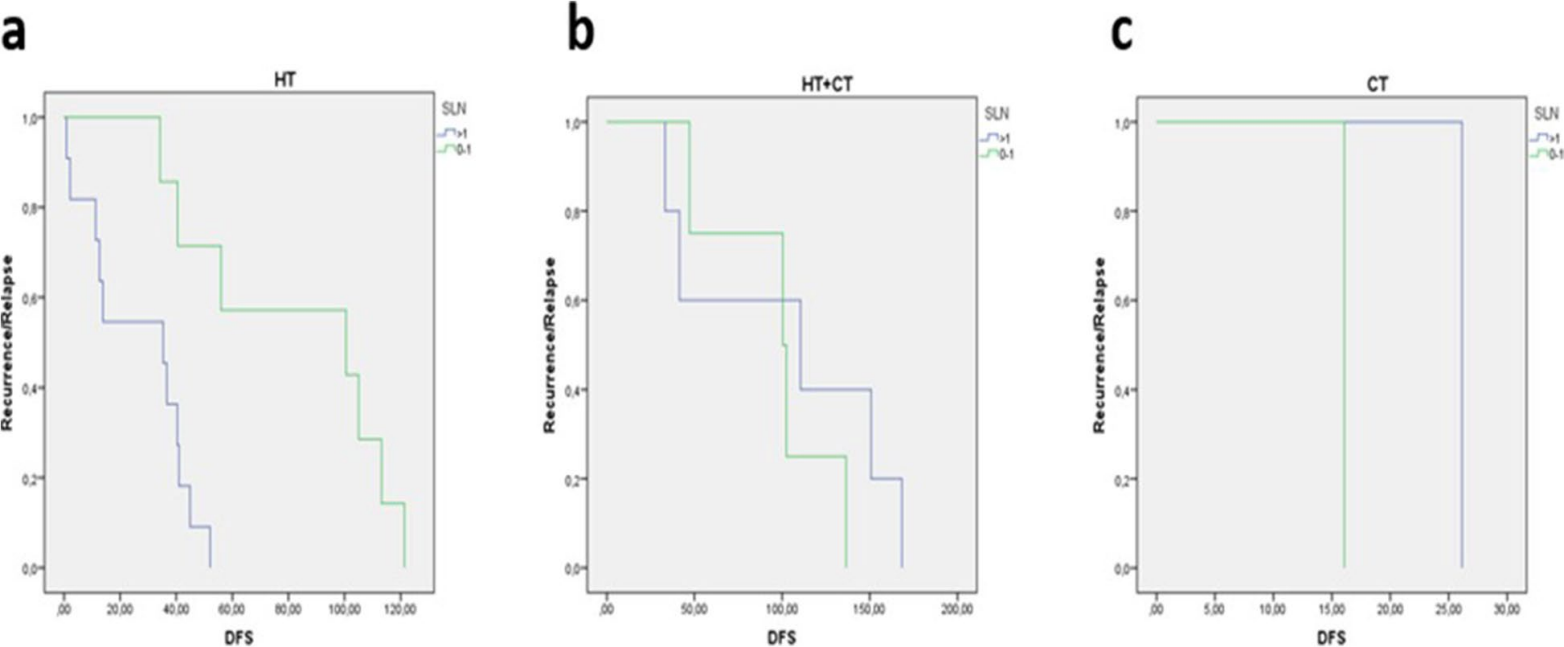
Kaplan–Meier showing median disease-free survival results according to sentinel lymph nodes and different treatment options

We planned a subgroup analysis according to different systemic therapy. In our early breast cancer population, a statistically significant difference in mDFS was showed for HT-treated patients who were SLN = 1 if compared to SLN > 1 (100.6 months versus 35.3 months). No relevant differences were retrieved in HT + CT (132.8 vs 154.9) or only CT (16.1 vs 26.1) subgroups (Fig. 3).

## Discussion

SLNB remains to this day the standard technique for staging the axillary cavity in patients with early-stage breast cancer [20–23]. It should be noted, despite a good lymph node collection, patients are still exposed to the risk of local or distant recurrence. Previous experiences also suggest that certain characteristics such as multifocal disease or parameter T can modify both the risk of lymph node involvement of the axillar and the risk of disease recurrence (both local and distant) [24]. The results of our study first confirm that the T parameter influences the appearance of RRs (T1 = 1.8%; T2 2.4%, T3 = 10.5% - chi-square 12.9; *p* = 0.002). Specifically, the study that enrolled a large cohort of patients showed that there is probably no direct relationship between the number of lymph nodes removed during SLNB and better DFS. In this regard, previous studies have shown that the risk of lymph node recurrence after negative SLNB is around 0–1.5% after a follow-up of at least 2 years [25].

Adjuvant therapies are among the factors that can influence the incidence of regional recurrences in patients with negative SLN [26–30]. Some randomized clinical trials have shown a positive effect of adjuvant therapies on the risk of loco-regional recurrence [31, 32]. Van Maaren et al. observed a lower risk or regional recurrence after breast-conserving surgery than after total mastectomy, suggesting a positive effect of radiotherapy on the regional recurrence rate [33]. In our study, a statistically significant difference in mDFS was showed for HT-treated patients who were SLN = 1 if compared to SLN > 1 (100.6 months versus 35.3 months). Instead, no relevant differences were retrieved in HT + CT (132.8 vs 154.9) or only CT (16.1 vs 26.1) subgroups.

In our experience, the local recurrence rate (breast and lymph nodes) in patients with negative sentinel lymph nodes was overall 1.8% (breast 0.3%; lymph nodes 1.5%) at a median follow-up of 44.2 months. Our results can therefore be considered in line with what is reported in the literature. These interesting results were obtained probably also thanks to the great care in limiting margins during conservative surgery. Besides, there was no significant difference between the relapse rate in those patients who had only one lymph node removed and those who had removed >1 node (2.11% vs 1.61%). Similar results were obtained for distant relapses. Other experiences have suggested that the distant relapse rate in this patient setting is around 3.5% [14]. Our series reported a lower percentage of distant relapses around 0.8% (0.6% visceral; 0.2% bone). A probable explanation for this discrepancy could be linked to the higher proportion of patients with favorable prognostic characteristics (ER +, PR +, HER2−, ki67 <20%) undergoing treatment with hormone therapy who developed indolent metastases mainly in the bone.

Limits of our study include the retrospective design and the presence of missing data that influence patient exclusion from the analysis (17%) mostly due to incomplete non-electronic medical records. Furthermore, it is a single-center experience, in which the outcome of the SLN > 1 group could be influenced by the progressive time-dependent improvement of the surgical technique, limiting the gap. Besides, to evaluate procedure cost-effectiveness, no available data from our clinical were useful to compare the incidence of peri- or postoperative adverse events incidence between groups.

In conclusion, our results confirm the low rate of breast cancer recurrence after conservative surgery if the primary tumor is completely excised with care for negative margins. Also, SNLB is to be considered as the standard of care for cN0 breast cancer patients even though there is likely a relationship between the number of resected SNL and mDFS. Our results showed no relevant difference in median DFS (64.9 vs 41.4) for SLN = 1 vs SLN > 1 group (*HR* 0.76, *CI 95%* 0.39–1.46; *p* = 0.38), except for a subset of the patients treated with hormone therapy. Further studies also involving molecular characteristics are needed.

## Abbreviations

SLNB: Sentinel lymph node biopsy
ALND: Axillary lymph node dissection
DFS: Disease-free survival
OS: Overall survival
RR: Regional recurrence
FNR: False-negative rate
FS: Frozen section
HT: Hormone therapy
CT: Chemotherapy
DM: Distant metastases
